# Transitioning from efgartigimod to tacrolimus in chronic inflammatory demyelinating polyneuropathy: a prospective case series

**DOI:** 10.3389/fimmu.2026.1916815

**Published:** 2026-09-15

**Authors:** Yujia Shen, Jianian Hu, Chong Sun, Yiyun Huang, Heting Cai, Kuan Liu, Xinyue Huang, Jian Sun, Yanyin Zhao, Chongbo Zhao, Jie Lin

**Affiliations:** 1 Department of Neurology, Huashan Hospital, Fudan University, Shanghai, China; 2 National Center for Neurological Disorders, Fudan University, Shanghai, China; 3 Huashan Rare Disease Center, Shanghai Medical College, Fudan University, Shanghai, China

**Keywords:** chronic inflammatory demyelinating polyneuropathy, efgartigimod, maintenance therapy, tacrolimus, treatment outcome

## Abstract

To describe the clinical outcomes of transitioning from efgartigimod to tacrolimus in chronic inflammatory demyelinating polyneuropathy (CIDP) patients. Six patients who completed the ADHERE trial and maintained clinical stability during the open-label extension phase (≥30 weeks of weekly efgartigimod) were transitioned to oral tacrolimus following efgartigimod discontinuation and were followed at 3-month intervals for 12 months. Clinical effectiveness was assessed utilizing the Inflammatory Neuropathy Cause and Treatment (INCAT) disability scale, centile Inflammatory Rasch-built Overall Disability Scale (cI-RODS), Medical Research Council (MRC) sum score (0-60), grip strength, 3m-Timed Up and Go Test (TUG) as well as electrophysiological examination. Safety was evaluated by monitoring adverse events. Peripheral T and B lymphocytes, interleukins and immunoglobulin levels were measured at each visit, and tacrolimus trough levels were monitored during follow-up. Three of six patients completed 12-month follow-up and achieved clinical stability or improvement by month 12. Two discontinued within three months due to poor clinical response and regained improvement following transition back to intravenous immunoglobulin or efgartigimod. One expressed a preference for traditional Chinese medicine and was lost to follow-up. No severe adverse events were observed. Oral tacrolimus may be a potential maintenance approach for sustaining clinical stability in a subset of CIDP patients stabilized on efgartigimod. Longitudinal naive B-cell monitoring warrants further investigation as a potential early indicator of clinical instability during tacrolimus maintenance therapy.

## 1 Introduction

Chronic inflammatory demyelinating polyneuropathy (CIDP) is an immune-mediated peripheral neuropathy characterized by progressive or relapsing sensorimotor dysfunction that can lead to severe disability in the absence of effective treatment (1). First-line therapies including steroids, intravenous immunoglobulin (IVIG) and plasma exchange are each effective in about two-thirds of patients (2) while approximately 30% of patients remain refractory. More recently, advances in targeted immunotherapy have expanded treatment options for CIDP patients. In the ADHERE study, subcutaneous neonatal Fc receptor (FcRn) inhibitor efgartigimod demonstrated clear efficacy in reducing pathogenic IgG levels and improving clinical outcomes. Subsequent open-label extension (OLE) data have further validated its durability of effect (3). However, several practical limitations constrain its real-world application. In China, efgartigimod has not yet been covered by public insurance for CIDP and thus carries a substantial economic burden, rendering long-term treatment financially prohibitive for many patients. Critically, there are currently no established evidence-based guidelines addressing the optimal maintenance strategy following efgartigimod discontinuation. To our knowledge, no prospective data currently exist to guide this clinical transition.

Accumulating evidence suggests that T-cells contribute to the pathogenesis of CIDP. Activated T cells can infiltrate peripheral nerves and nerve roots, disrupt the blood-nerve barrier, and further release pro-inflammatory cytokines, resulting in nerve damage (4). Tacrolimus is an oral calcineurin inhibitor that suppresses T-cell activation through forming a complex with FK506-binding protein 12 (FKBP12), leading to inhibition of calcineurin-mediated activation of nuclear factor of activated T cells (NFAT) and subsequent reduction of IL-2-dependent T-cell proliferation (5). It has been widely used in various autoimmune diseases and has been explored as a therapeutic option in immune-mediated neuropathies (6). Previous studies have demonstrated that tacrolimus improves outcomes in patients with relapsing-remitting CIDP, and may reduce relapse rates while decreasing corticosteroid dependence (7). Yet, it has also been reported to cause neurotoxicity, including CIDP-like syndromes in rare cases, particularly in organ-transplant populations receiving long-term immunosuppression (8).

In the present study, we evaluated the clinical outcomes of transitioning from efgartigimod to tacrolimus in a case series of six CIDP patients and explored associated immunological changes.

## 2 Methods

### 2.1 Study design

This prospective case series was conducted in Huashan Hospital, Fudan University from February 2025 to February 2026. All six CIDP patients were previously enrolled in and completed the ADHERE study, where they received weekly 1000 mg subcutaneous efgartigimod PH20, and achieved a robust clinical response followed by maintained clinical stability over at least 30 weeks of weekly efgartigimod administration during the open-label extension (OLE) phase. Clinical stability was defined as the absence of protocol-defined clinical deterioration, namely, neither ≥ 1-point increase in the aINCAT score at a consecutive visit nor ≥ 2-point increase at a single visit relative to the open-lable phase baseline. All participants were transitioned to tacrolimus following the cessation of efgartigimod. Baseline demographic and clinical characteristics of all patients were collected at the time of treatment switch. Cell-based assay was performed for screening of anti-paranodal antibodies (9). The study was performed in accordance with the Declaration of Helsinki and approved by the Ethics Committee of Huashan Hospital, Fudan University. Written informed consent was obtained from all participants.

### 2.2 Treatment

Following efgartigimod discontinuation, tacrolimus was administered orally at an initial dosage of 3 mg/day and further individualized to achieve a target trough concentration of 5–10 ng/mL. In a subset of patients, a proprietary *Schisandra sphenanthera* ethanol-extract preparation (Wuzhi capsule) was co-administered as an adjuvant. It interacts with tacrolimus through inhibition of CYP3A4/5 and p-glycoprotein so as to slow down degradation and increase intestinal absorption while reducing biliary excretion, thereby raising tacrolimus blood concentrations (10).

### 2.3 Clinical assessments

Patients were assessed at baseline and at months 3, 6, 9 and 12. Clinical evaluations at each visit included the Inflammatory Neuropathy Cause and Treatment disability score (INCAT), centile Inflammatory Rasch-built Overall Disability Scale (cI-RODS), Medical Research Council (MRC) sum score (0-60), grip strength (Martin vigorimeter), and the 3m-Timed Up and Go Test (TUG). Electrophysiological examinations were also performed at baseline, month 6 and month 12 to further support the assessment of clinical efficacy.

In addition to the scheduled clinical assessments mentioned above, patients were instructed to report any perceived worsening of neurological symptoms and underwent additional clinical evaluation when clinically indicated. Poor response to tacrolimus was defined as patient-reported clinical worsening accompanied by an objective deterioration (≥ 1-point increase) in the aINCAT score.

### 2.4 Laboratory assessments

Peripheral blood samples were collected in EDTA-containing tubes and processed according to the standard protocol at each visit (11). Serum and plasma samples were used to assess liver function tests, tacrolimus trough concentrations, serum immunoglobulin levels, and serum interleukin levels. Concurrently, peripheral blood mononuclear cells (PBMCs) were isolated for immunophenotyping by flow cytometry to analyze T and B lymphocytes and their subtypes. T cell populations included total T cells (CD3+), helper T cells (Th, CD4+), cytotoxic T cells (Tc, CD8+), and double-positive T cells (CD3+CD4+CD8+). Activated T cell subsets were identified as CD4+HLA-DR+ and CD8+HLA-DR+ cells. Naïve and memory subsets of CD4+ and CD8+ T cells were further analyzed, including naïve, central memory, effector memory, and effector cells. Functional CD4+ T cell subsets included Th1, Th2 and Th17 cells. B lymphocytes and their subsets were analyzed using a similar strategy, including CD20+ B cells, transitional B cells, plasmablasts, memory B cells, naïve B cells, and B1a cells.

### 2.5 Outcome measures

The primary outcome was clinical stability or improvement at month 12. Clinical improvement was defined as meeting at least one of the following criteria: a reduction of ≥ 1 point on the adjusted INCAT scale or an increase of ≥ 4 points on the cI-RODS while clinical deterioration was defined as an increase of >1 point on INCAT or a decrease of ≥ 4 points on cI-RODS from baseline. Otherwise, patients were considered clinically stable. Secondary outcomes included time to first clinical response, changes in grip strength, TUG performance, MRC sum score, electrophysiological parameters, and longitudinal immunophenotypic profiles. Adverse events and treatment discontinuations were recorded throughout follow-up.

### 2.6 Statistical analysis

Continuous variables were presented as mean ± standard deviation (SD) or median (range). No formal hypothesis testing or adjustment for multiple comparisons was performed. All statistical analyses and graphical visualizations were performed using R (version 4.6.0; R Foundation for Statistical Computing, Vienna, Austria).

## 3 Results

### 3.1 Demographic and baseline clinical characteristics

All patients fulfilled the 2010 EFNS/PNS criteria for definite CIDP. Baseline was defined as the time of therapy switching. Median age at disease onset was 31.8 years (range, 22.5-60.8). Median age at baseline was 39.3 years (range, 32.6-65.6). Median disease duration at baseline was 5.1 years (range, 3.1-10.6). All six patients achieved clinical improvement following efgartigimod treatment, with median INCAT scoring 1 (range, 0-5) and median cI-RODS scoring 79.5 (range, 53-100). On electrophysiological examination, five showed demyelinating features, including four accompanied by axonal involvement. One patient presented no significant electrophysiological abnormalities. Notably, two patients tested positive for anti-Neurofascin-155 (NF155) antibody, another one positive for anti-contactin-associated protein 1 (Caspr 1) antibody while other three patients tested negative for all anti-paranodal antibodies. Demographic and baseline clinical characteristics of the six patients (four males and two females) are presented in **Table 1**.

**Table 1:** Baseline demographic and clinical characteristics of all enrolled patients.

Baseline characteristic	Pt1	Pt2	Pt3	Pt4	Pt5	Pt6
Gender	Female	Male	Male	Male	Female	Male
Age at onset (years)	32.3	30.5	31.2	22.5	55.4	60.8
Age at transition (years)	42.8	35.8	34.3	32.6	59.2	65.6
Disease Duration at transition(years)	10.6	5.3	3.1	10.1	3.8	4.8
Baseline clinical status						
INCAT	1	3	1	1	0	5
cI-RODS	71	53	100	88	88	55
Grip strength (Left)(kPa)	69	108	108	67	105	0
Grip strength (Right)(kPa)	77	107	120	73	103	20
Electrophysiological abnormalities	Sensorimotor demyelinating polyneuropathy with axonal damage	Sensorimotor demyelinating polyneuropathy with axonal damage	No significant electrophysiological abnormalities	Sensorimotor demyelinating polyneuropathy with axonal damage	Sensorimotor demyelinating polyneuropathy	Sensorimotor demyelinating polyneuropathy with axonal damage
Anti-paranodal antibody	Caspr1(+)	NF155(+)	(-)	NF155(+)	(-)	(-)

INCAT, Inflammatory Neuropathy Cause and Treatment; cI-RODS, centile Inflammatory Rasch-built Overall Disability Scale; Caspr 1, Contactin-associated protein 1; NF155, Neurofascin 155.

### 3.2 Treatment completion and tolerability

Three patients adhered to the prescribed regimen throughout follow-up. The Wuzhi capsule was co-administered in all three patients. The mean tacrolimus trough concentration in the three patients was 7.18 ± 0.31 ng/mL by month 12. No clinically significant hepatotoxicity was observed.

Another two patients showed poor clinical response within three months. Specifically, Patient 4 presented with worsening weakness, with the INCAT score increasing from 0 + 1 at enrollment to 2 + 1 (total score, 1 to 3). Patient 5 reported worsening numbness in both hands and increased weakness in all four limbs, with the INCAT score increasing from 0 + 0 at enrollment to 4 + 5 (total score, 0 to 9). Both were subsequently switched to intravenous immunoglobulin (IVIG) or efgartigimod and regained clinical improvements. One patient discontinued tacrolimus due to personal preferences for Chinese traditional medicine and was lost to follow up, with subsequent outcome unknown.

### 3.3 Clinical outcomes

Among the three responders, all reached an INCAT total score of 0 at month 12. Patients 1 and 3 were considered clinically stable, with total INCAT scores decreasing from 1 to 0. Both changes occurred in the upper-limb and thus were not regarded as improvement on the adjusted INCAT scale. In contrast, Patient 2 showed overall improvement by month 12, with the total INCAT score decreasing from 3 to 0.

Time to first clinical response differed across patients. Patient 3 first achieved an INCAT score of 0 at month 3, while Patient 1 reached this threshold at month 9; both maintained this status throughout the remainder of follow-up. This clinical course was supported by other assessments. Patient 1’s cI-RODS score increased from 71 to 88 by month 3 and remained steady, while Patient 3 maintained a maximal score of 100 from baseline. Similarly, MRC scores and TUG performance remained consistent in both patients, while grip strength showed minor, non-directional fluctuations.

Patient 2 showed a more fluctuating clinical course. Although the INCAT score remained stable during the first six months, it rose transiently to 4 at month 9, coinciding with a reported episode of alcohol consumption. This temporary shift was also reflected in grip strength and TUG performance. Nevertheless, these objective markers recovered by month 12, with the INCAT score returning to 0 and cI-RODS showing progressive improvement throughout follow-up (**Figure 1**).

**Figure 1:**
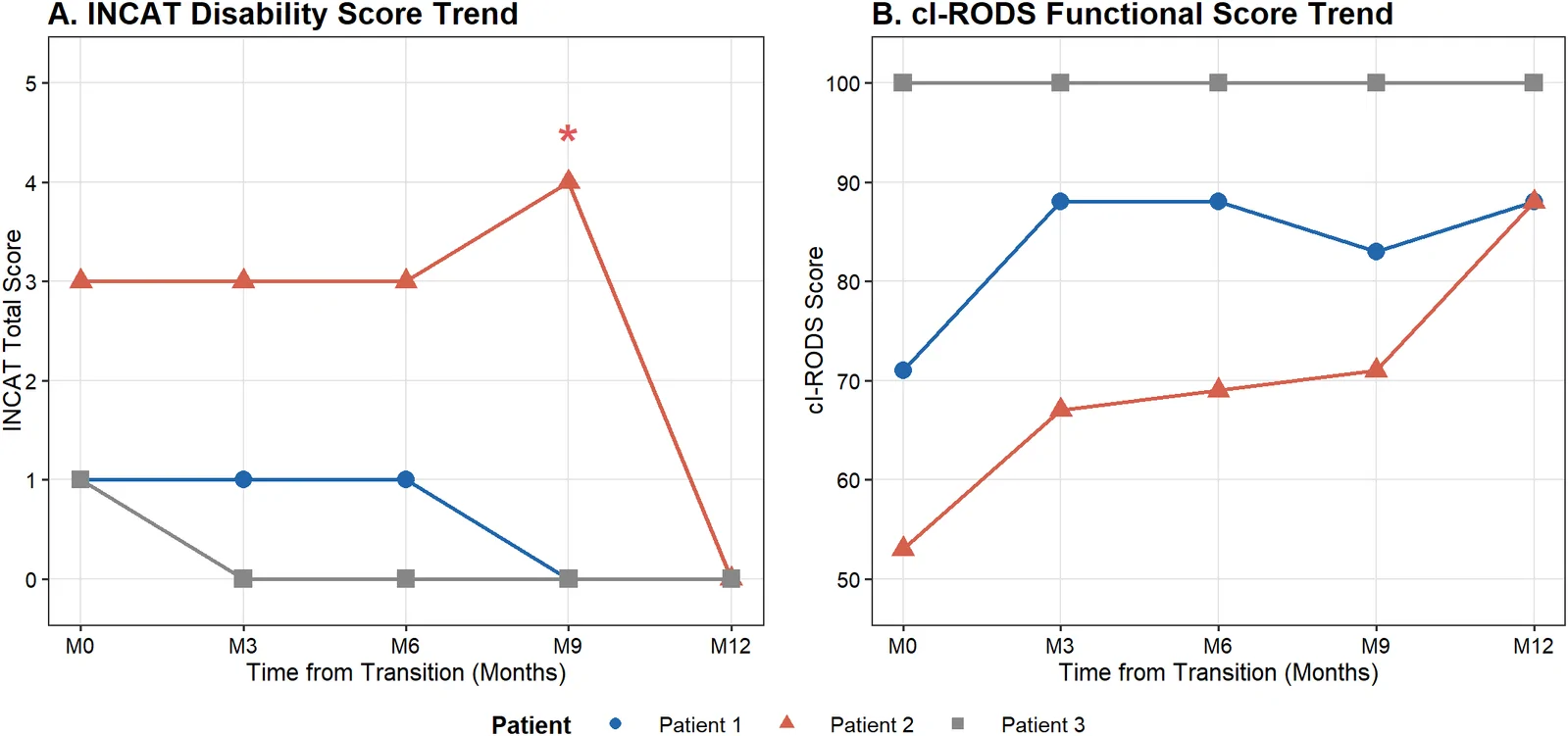
Longitudinal trends of clinical metrics in three patients. Individual trends of **(A)** INCAT disability scores and **(B)** cI-RODS functional scores are shown from baseline (M0) to 12 months (M12). Red asterisk in **(A)** indicates a transient increase in disability for Patient 2 at M9. INCAT, inflammatory neuropathy cause and treatment; cI-RODS, centile Inflammatory Rasch-built overall disability scale; M, Month.

Electrophysiological assessments further characterized the clinical changes. By month 6, Patients 1 and 2 showed improvement in two motor nerves, while minor declines were noted in several others. By month 12, both patients demonstrated favorable electrophysiological changes. Patient 1 showed increased motor and sensory amplitudes, improved conduction velocities in selected motor nerve segments, and shortening of the ulnar F-wave latency. Patient 2 showed more pronounced improvement, including shorter distal and F-wave latencies, faster motor conduction velocities, increased CMAP amplitudes, and reappearance of previously absent tibial F-wave and peroneal motor responses. Patient 3 remained electrophysiologically stable throughout the follow-up period, without evidence of significant polyneuropathic changes.

### 3.4 Safety of tacrolimus post treatment transition

Serum IgG levels were within safe physiological ranges throughout the follow-up in all patients. Liver function was also monitored every six months. Patient 3 showed transiently elevated liver enzymes, which required no intervention and were not classified as an adverse event after hepatological review. Additionally, monitoring of C3, C4, and CH50 revealed no signs of systemic complement depletion or hyperactivation.

### 3.5 Effects of tacrolimus on lymphocytes

Peripheral blood lymphocyte immunophenotyping by flow cytometry was longitudinally performed in the three patients who completed full follow-up, revealing dynamic changes in both T- and B-cell compartments.

#### 3.5.1 T-cell subtype dynamics

T-cell subsets presented relatively consistent trends across three patients. Double-positive (CD3+CD4+CD8+) T cell proportions decreased steadily from baseline levels of 0.28%-3.49% to 0.11% or below by month 12, consistent with progressive suppression of aberrantly activated T-cell populations under tacrolimus. Activated Th cell (CD3+CD4+DR+) proportions showed a shared initial increase at month 6, reaching 45.02%–54.06%, followed by a decline to levels between 5.97% and 8.32% by the end of the study. Similar trends were noted for activated Tc cells (CD3+CD8+DR+) proportions in Patient 2, whereas Patients 1 and 3 demonstrated a more progressive decline throughout follow-up. The Th1/Th17 ratio remained within the normal range across all patients throughout follow-up, with no sustained skewing toward a pro-inflammatory phenotype, despite some interindividual and intraindividual fluctuation (**Figure 2**).

**Figure 2:**
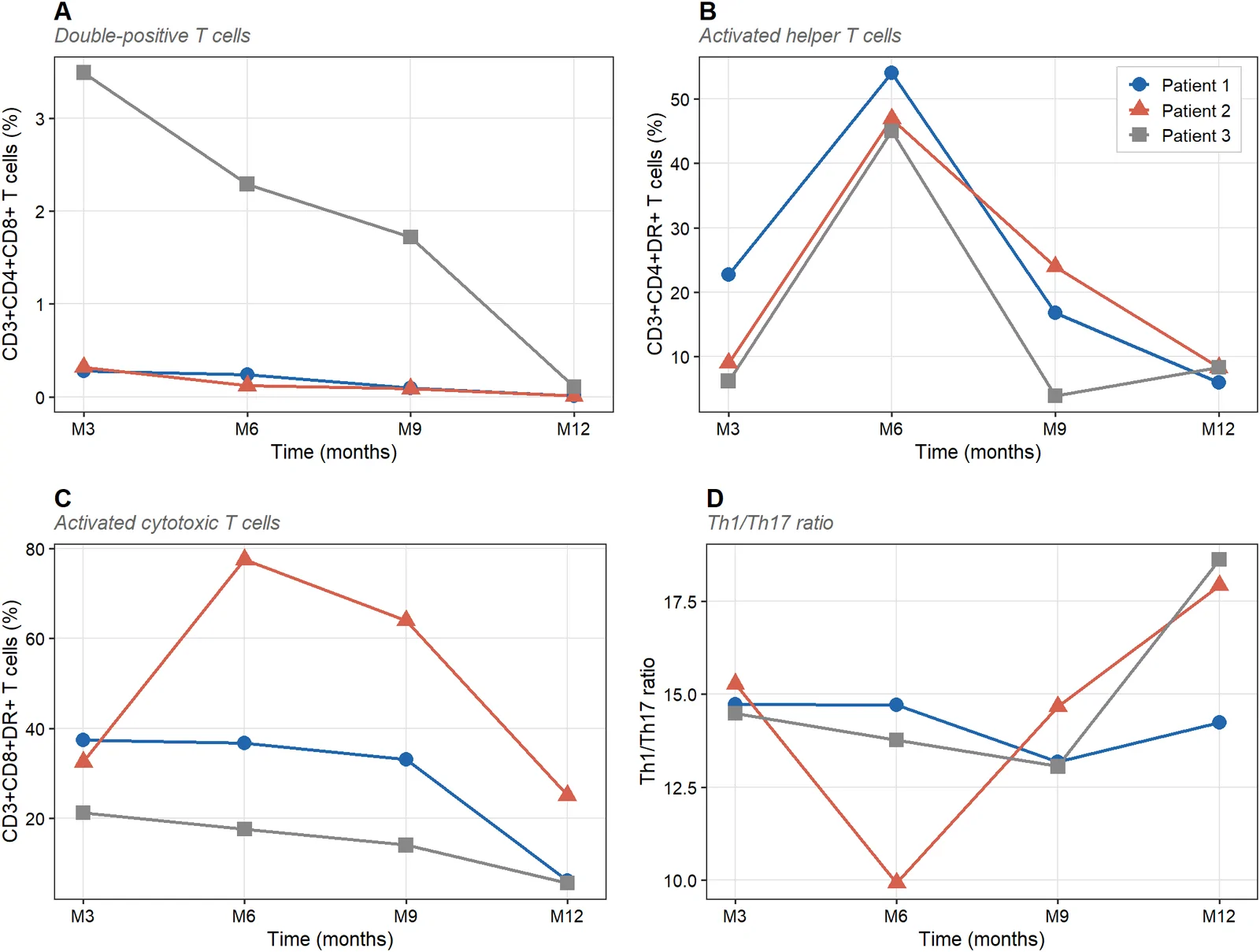
Longitudinal T-cell subset dynamics during tacrolimus maintenance therapy. **(A)** Double-positive (CD3+CD4+CD8+) T-cell proportions. **(B)** Activated helper T-cell (CD3+CD4+DR+) proportions. **(C)** Activated cytotoxic T-cell (CD3+CD8+DR+) proportions. **(D)** Th1/Th17 ratio. The data represent longitudinal observations from the three patients who completed follow-up. No inferential statistical testing was performed; changes over time should therefore be interpreted as descriptive individual-patient trends. M, month.

#### 3.5.2 B-cell subtype dynamics

In contrast, B-cell subsets exhibited pronounced interindividual variability during the first nine months of follow-up, followed by a convergence toward a more uniform profile by month 12. Patient 1 maintained largely stable B-cell proportions throughout follow-up. Patient 3 demonstrated a transient reduction in circulating CD20+ B cells at month 6 in the absence of any identifiable clinical trigger, yet remained clinically stable.

Patient 2 demonstrated the most marked changes, with the naive B-cell fraction rising progressively from month 3 and peaking at 90.27% at month 9, concurrent with transient clinical fluctuations. Importantly, this rise preceded the observed INCAT worsening by approximately six months, raising the possibility that naive B-cell expansion may serve as an early immunological signal of impending clinical instability. Following alcohol cessation, both the clinical and immunological parameters normalized by month 12.

By the end of follow-up, B-cell profiles had aligned across all patients. Both CD27− memory B cells and switched memory B cells expanded relative to mid-study levels, while the B1a subset, which had declined during the first half of follow-up, recovered to baseline values (**Figure 3**).

**Figure 3:**
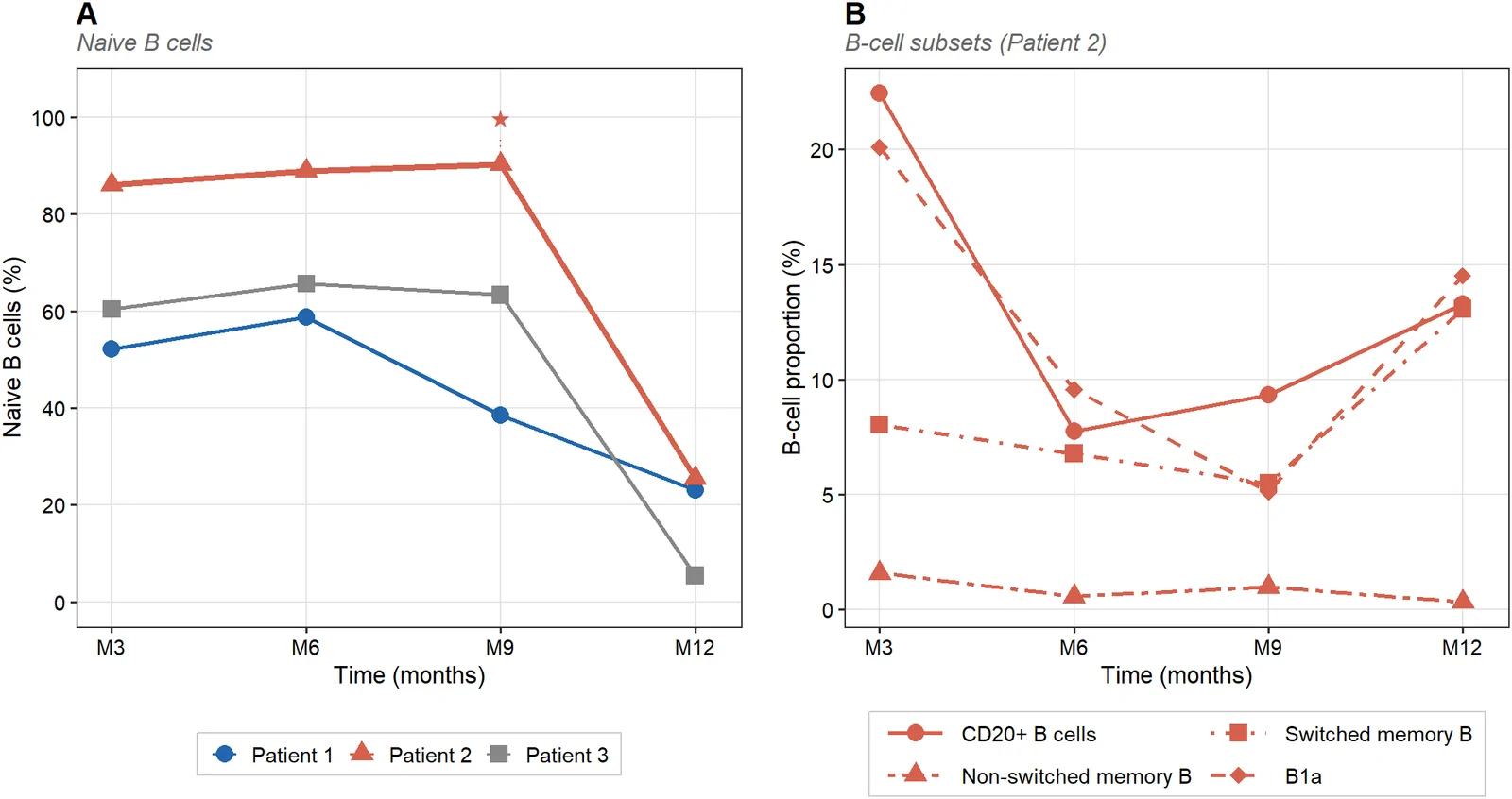
Longitudinal B-cell subset dynamics during tacrolimus maintenance therapy. **(A)** Naive B-cell proportions in all three patients across the 12-month follow-up. **(B)** B-cell subset dynamics in Patient 2, including CD20+ B cells, non-switched memory B cells, switched memory B cells, and B1a cells. The star indicates alcohol consumption event at month 9. No inferential statistical testing was performed, and the longitudinal changes should be interpreted as observational individual-patient trends. M, month.

## 4 Discussion

### 4.1 Tacrolimus as maintenance therapy following efgartigimod

In this study, we prospectively evaluated the clinical outcomes of transitioning from efgartigimod to oral tacrolimus as maintenance therapy in clinically stable CIDP patients. Among the six participants, three completed the 12-month follow-up and demonstrated clinical stability or improvement by month 12, as measured by INCAT, cI-RODS, grip strength, TUG and electrophysiological examination. In both cases of poor response, withdrawal occurred within the first three months, and clinical improvement was regained with conventional therapies. This early and reversible failure suggests that close monitoring at the early stage of transition may serve as a practical framework to identify patients unlikely to benefit from the strategy.

Also, we observed that clinical score improvements or stability were achieved preceding electrophysiology parameters. This dissociation is consistent with the classical pathophysiology of CIDP, where functional recovery often outpaces structural remyelination (12), and suggests that clinical manifestations remain the more sensitive and responsive indicators of disease status during maintenance therapy.

### 4.2 The transitional window and early clinical fluctuation

A transient reduction in grip strength was observed at month 3 in all three patients, despite stable INCAT and cI-RODS scores. We interpret this as a pharmacological transitional window following efgartigimod withdrawal. As FcRn blockade is discontinued, circulating IgG levels consequently recover, while tacrolimus requires several weeks to reach stable drug trough (13, 14). The resulting gap in immune coverage is further supported as the peak in activated helper T-cell proportions was also observed at month 6 across all three patients. Taken together, these suggest that the early stage of transition may represent a period of particular vulnerability, during which clinical monitoring, or a possible bridging strategy may be warranted.

### 4.3 Implications for autoimmune nodopathy subtypes

Two of the three patients who completed follow-up were tested positive for paranodal antibodies, Patient 1 for CASPR1-IgG and Patient 2 for NF155-IgG. Both met criteria for autoimmune nodopathy according to the 2021 EAN/PNS guidelines. While Patient 1 maintained clinical stability throughout follow-up, Patient 2 demonstrated a more complicated course. NF155-IgG4 production depends on sustained T follicular helper (Tfh) cell activity, and tacrolimus-mediated calcineurin inhibition may attenuate Tfh-driven IgG4 (15). Serial anti-NF155 IgG titers remained stable at 1:100 from baseline through month 12, with titers of 1:200 and above consistently negative. The transient clinical deterioration at month 9 thus occurred interestingly against a background of unchanged antibody titer. Whether T-cell-mediated suppression of the Tfh–B cell axis can sustain durable disease control in NF155-AN without driving antibody eradication remains an open question, but the clinical course of this patient offers preliminary grounds for cautious optimism.

### 4.4 Naïve B-cell expansion as a potential early warning signal

As previously mentioned, naive B-cell proportion rose progressively from month 3, peaking at 90.27% at month 9, concurrent with clinical deterioration. This temporal lead raises the possibility that longitudinal naive B-cell monitoring could serve as an early warning signal of impending instability during tacrolimus maintenance. The normalization of naive B-cell proportions alongside clinical recovery by month 12, without any change in tacrolimus dosing, further supports that this expansion might reflect a reversible perturbation rather than a fundamental failure of the maintenance strategy. It is also worth considering whether the potential utility of naive B-cell monitoring as an early warning signal may be particularly relevant in NF155-IgG4-positive AN, given the mechanistic dependence of IgG4 production on Tfh-driven B-cell activity. Whether a similar pattern holds in classical CIDP or other AN subtypes remains an open question that prospective studies with larger, subtype-stratified cohorts will be needed to further address.

### 4.5 INCAT and CI-RODS dissociation

An intriguing dissociation was observed between INCAT scores and cI-RODS in patient 2. While the INCAT score rose transiently at month 9 and subsequently normalized, cI-RODS presented a progressive improvement throughout the entire 12 months. We suspect this dissociation may partly reflect the pharmacological properties of tacrolimus. As calcineurin plays a role in skeletal muscle excitation-contraction coupling through its regulation of ryanodine receptor (RyR) and sarco/endoplasmic reticulum Ca^2+^-ATPase (SERCA) activity, tacrolimus-mediated calcineurin inhibition may therefore exert a direct, immune-independent effect on muscle contractile efficiency that is captured by examiner-rated motor assessments such as INCAT but less reflected in patient-reported functional scales (16, 17). In other words, the progressive improvement in cI-RODS throughout follow-up—including during the month 9 episode, may more faithfully represent the sustained immunological benefit of tacrolimus maintenance, while the transient INCAT fluctuation reflects a composite signal in which drug pharmacology and disease activity are not fully separable. This may further highlight the necessity of multidimensional assessment in CIDP patients, especially during episodes of clinical fluctuation.

### 4.6 Clinical implications and generalizability

Given the exploratory design and limited sample size of this study, it is not yet possible to define a specific subgroup most likely to benefit from tacrolimus maintenance therapy. Nevertheless, our findings suggest a potential clinical phenotype warranting further investigation: patients who have achieved a robust and sustained clinical response to efgartigimod and whose clinical stability is not rapidly lost during dose-interval extension or withdrawal while tacrolimus is initiated. This strategy may be particularly relevant when continued long-term efgartigimod treatment is constrained by cost or accessibility. Close clinical monitoring remains essential, particularly during the first three months, as two patients in our cohort experienced clinical deterioration during this period and required tacrolimus discontinuation and a return to other therapies.

Reliable predictors of a successful transition have not yet been identified. Although patients with a clearly defined antibody-mediated disease mechanism may be expected to respond to FcRn blockade, it remains uncertain whether they are more dependent on continued efgartigimod or more likely to benefit from subsequent tacrolimus maintenance. In our cohort, patients with nodal/paranodal antibodies were represented among both those who completed follow-up and those who discontinued tacrolimus, precluding conclusions regarding the predictive value of antibody status. Larger prospective studies stratified by clinical phenotype and antibody status are needed to evaluate the predictive value of pre-transition clinical characteristics, the magnitude and durability of the response to efgartigimod, maintenance of clinical stability during dose-interval extension, and humoral and cellular immune biomarkers, and ultimately to establish practical criteria for patient selection.

### 4.7 Limitations

Several limitations should be acknowledged. First, the small sample size inherent to this first-in-field pilot dataset necessarily limits statistical power and generalizability, particularly regarding the subgroup analysis of antibody-positive patients. Nonetheless, this constitutes the only prospective evidence currently available to inform maintenance strategy decisions following efgartigimod stabilization. Second, the high dropout rate (50%) introduces a potential survival bias toward responders in the longitudinal analysis. Third, though immunological associations were identified, the 12-month follow-up period may not capture long-term relapse risks or cumulative toxicity. Continued longitudinal monitoring of these patients will help to further characterize treatment response and inform the feasibility of larger prospective studies.

## 5 Conclusion

To our knowledge, this is the first prospective case series to address maintenance strategy following efgartigimod stabilization in CIDP, and provides preliminary evidence that oral tacrolimus may represent a feasible option for selected patients. Among patients who completed 12-month follow-up, clinical stability was maintained and tacrolimus trough concentrations were consistently within target range, with no significant drug-related toxicity. The 50% dropout rate in the overall cohort highlights the importance of patient selection and early response monitoring, with the first three months appearing to represent a critical decision window.

The immunological data, while exploratory, suggest that longitudinal B-cell subset profiling—particularly monitoring of the naive B-cell fraction may offer clinically actionable early warning information during maintenance therapy, warranting prospective validation in larger, subtype-stratified cohorts. Taken together, these findings support the rationale for larger controlled studies evaluating tacrolimus as a maintenance option in CIDP, with particular attention to the transitional period following efgartigimod discontinuation and the potential utility of immune monitoring in guiding clinical decisions.

## Data Availability

The original contributions presented in the study are included in the article/supplementary material. Further inquiries can be directed to the corresponding authors.
